# Responsibility of the Individual Dentist Towards the State Organization

**Published:** 1906-05

**Authors:** W. C. Miller

**Affiliations:** Augusta, Ga.


### RESPONSIBILITY OF THE INDIVIDUAL DENTIST TOWARDS THE STATE ORGANIZATION.

By W. C. Miller, D. D. S., Augusta, Ga.

At this particular season of the year, a feeble effort to say something on the above topic may be of some interest to the profession, if perchance it will add to the attendance, one man who would not have gone, had not the writer made this effort, he will feel fully compensated. The state organizations are supposed to be composed of the men in the profession who would be found on the side that stands for everything tending to uphold the dignity and glory of dentistry. I am glad I used that word "supposed." This will strike a vibrating chord within that man's breast, who remains at home, and says that the majority of the members of the State Organization, are no better than the "parlor man." This is not the cry only of such bodies, but we all know that many men are out of the churches to-day because they feel that they are much better than the average church member. Sad state of affairs.

Must I stay at home and be content with my own little selfish world, just because some other fellow goes who has no business there? I shall contend that every ethical man is in part responsible for the success of his state meeting, and further, that when he stays at home, he is responsible for the lack of professional courtesy that exists between the better men in the profession.

Some of our best dentists are out of our organizations, and I expect will remain out, until they from the *outside*, by "backbitings and vile accusations" of those on the *inside* will have purged the thing, and made it a place fit to hold only the pure in heart. I venture there are more men out of the organizations who accuse more men in the organizations, of insincerity, lack of ethical dignity and everything that it takes to make a gentleman, than there are in the organizations who find that this thing does not exist among themselves. This does not include any of the accusations that the "parlor man" might bring against us either. Now must a good man remain at home because he has heard his ethical brother say that he is not a fit member of the Dental Society? Who set him to be a judge among us?

I do not think there is a town of any consequence in Georgia, where the dentists of that town are entirely in harmony, and in a position to successfully carry on a small organization among themselves. To what may this be attributed? Can it not be answered by saying that the dentists of cities and towns do not as a *whole* attend the state meetings, and are not imbued with that feeling of good fellowship inculcated by such associations? We have men who are our superiors, and they should be recognized with kindness and condescension. We cannot do as well as they, but we may do our best, and in doing our best we will find our equals, to whom we may be courteous and affable, and our inferiors, who have not been blessed with our share of opportunities and skill, will be always obedient and submissive. I do not think that I am mistaken when I say, that this is the spirit that pervades any body of honest men, and is to be found among the organized dentists, yet we should always be on the watch that nothing beyond this ever enters our ranks. The man hampered by numberless limitations may not place matters before us just as we would like it, but he is honest and a gem of thought is there. The man far above us, a height that we know we can never reach is not a "political grafter" just because he is so much in evidence. That man is certainly a curiosity who attends the meeting, and goes there to contribute something for the welfare of the organization, as well as to be benefited by attending, and returns home feeling that he would have been better off had he not gone. In attending, I do not mean it in a passing sense, for the man who leaves his office the day before the meeting is called to order, listens to the president's address, and then absents himself to attend some ball game or to visit some club rooms, meet a friend at some depot, and then pops in to see what is going on occasionally, and get a badge for the banquet or barbecue, returning home after it is all over, *has not attended the meeting,* don't deceive yourself. As a rule we have only three days to attend to the business and hear the papers, enter into the discussion of same, attend the clinics, etc., etc. Now gentlemen, nothing should come up to detract at all from any of this, do what is to be done, if it consumes a whole week, don't let refreshments bother you, it is well enough to think about this after all of the business has been transacted. If we leave home to attend the meeting, let's attend it and see it all, and I know we all will be the better men by so doing. For myself, I care not what I may be called, and how inconsistent I may be accused of being, I am going to say and do just what I feel that a gentleman, who values his honor and professional dignity above dollars and cents, would say and do. In a speech made by Alexander Stephen's in 1855, we have these words: "I am afraid of nothing on earth, or above the earth, or under the earth, except to do wrong. The path of duty I shall ever endeavor to travel, fearing no evil and dreading no consequences." When this is the standard that we all set up, and by the grace of God endeavor to live up to, then we will have, not only better meetings, but a more united profession, and be better fitted to serve our fellow man, and lift the banner of such a noble calling from the mire in which some would have it trail, meriting in the end the plaudit of our Chief Demonstrator, "enter into the joys of thy Lord."

